# Intersections of Ubiquitin-Proteosome System and Autophagy in Promoting Growth of Glioblastoma Multiforme: Challenges and Opportunities

**DOI:** 10.3390/cells11244063

**Published:** 2022-12-15

**Authors:** Rhett Visintin, Swapan K. Ray

**Affiliations:** 1 Department of Chemistry and Biochemistry, University of South Carolina, Columbia, SC 29208, USA; 2 Department of Pathology, Microbiology and Immunology, University of South Carolina School of Medicine, Columbia, SC 29209, USA

**Keywords:** glioblastoma multiforme (GBM), ubiquitin-proteasome system (UPS), autophagy, crosstalk between the UPS and autophagy, GBM cell survival and proliferation, treatment resistance, new therapeutic opportunities, induction of apoptosis in GBM

## Abstract

Glioblastoma multiforme (GBM) is a brain tumor notorious for its propensity to recur after the standard treatments of surgical resection, ionizing radiation (IR), and temozolomide (TMZ). Combined with the acquired resistance to standard treatments and recurrence, GBM is an especially deadly malignancy with hardly any worthwhile treatment options. The treatment resistance of GBM is influenced, in large part, by the contributions from two main degradative pathways in eukaryotic cells: ubiquitin-proteasome system (UPS) and autophagy. These two systems influence GBM cell survival by removing and recycling cellular components that have been damaged by treatments, as well as by modulating metabolism and selective degradation of components of cell survival or cell death pathways. There has recently been a large amount of interest in potential cancer therapies involving modulation of UPS or autophagy pathways. There is significant crosstalk between the two systems that pose therapeutic challenges, including utilization of ubiquitin signaling, the degradation of components of one system by the other, and compensatory activation of autophagy in the case of proteasome inhibition for GBM cell survival and proliferation. There are several important regulatory nodes which have functions affecting both systems. There are various molecular components at the intersections of UPS and autophagy pathways that pose challenges but also show some new therapeutic opportunities for GBM. This review article aims to provide an overview of the recent advancements in research regarding the intersections of UPS and autophagy with relevance to finding novel GBM treatment opportunities, especially for combating GBM treatment resistance.

## 1. Introduction

Glioblastoma multiforme (GBM), often called just glioblastoma, is a deadly brain malignancy that is characterized by rapid and aggressive cell proliferation, diffuse tumor morphology, and radio-resistant and chemo-resistant recurrence after initial treatments. Due to these characteristics, GBM has a very poor prognosis, with current treatments leading to an average survival time of 15 months or less [1]. Among the central nervous system (CNS) malignancies it is both the most common and the deadliest, and thereby significant research has gone into developing new treatments [1]. Unfortunately, little progress has been made in producing therapies that make it past clinical trials; and thus, the standard course of treatment strategy involving surgical resection, ionizing radiation (IR), and the DNA alkylating drug temozolomide (TMZ) has remained essentially unchanged for the last few decades [2]. GBM has innumerable insidious traits, which impede development of new therapies, including the relative impermeability of the blood-brain barrier (BBB), the invasion of malignant cells into the brain parenchyma beyond the solid tumor border, the modification of the immune microenvironment by the GBM cells, and the presence of GBM stem cells (GSCs), which are resistant to IR and chemotherapy and lead to recurrence of the tumor after its initial treatments [2].

Cancer cell survival is an efficient choreography and dependent on coordinated anabolic and catabolic processes. Faced with limited resources, cancer cells must reuse and recycle components that are damaged or have outlived their usefulness. In addition, many proteins and organelles can be cytotoxic in themselves when allowed to accumulate unchecked. This resource management becomes even more tricky and critical when cells are subjected to environmental stresses; GBM cells face such stresses in the hypoxic regions within the tumor, as well as from the IR and chemotherapeutic agents used throughout the treatments [3,4]. The ability of GBM cells to survive and propagate despite these stresses is at least partially due to the presence of differential activities of the two main cellular ‘degradation and recycling’ mechanisms: ubiquitin-proteasome system (UPS) and autophagy [5,6]. The UPS gets involved in specific and limited amounts of degradation and recycling of the undesirable and useless proteins in the cells, while autophagy is activated mainly to execute bulk degradation of the damaged cellular components and organelles for generating new building blocks such as amino acids, nucleotides, and sugars for recycling and supporting cell survival. The UPS and autophagy also serve a dual purpose as post-translational regulators of biochemical pathways via, respectively, selective and bulk degradation of the damaged proteins [7,8]. Owing to their diverse, complimentary, and somewhat overlapping functions, there exists a rich network of regulatory relationships between these two recycling mechanisms, altogether exerting a significant effect on cellular stress responses and survival [9,10,11,12]. Therefore, when considering modulation of UPS or autophagy for the purpose of discovering new treatment opportunities in GBM, it may be helpful to consider the interrelations of these two degradative and recycling mechanisms as they occur in GBM.

Overall, the nature of interactions between UPS and autophagy relating to promoting cell survival or death in GBM remains understudied. Studies concerning one system, or the other are abundant, but rarely do they measure the impact on the complimentary components of these two highly interconnected systems. Therefore, interactions between these systems that may affect experimental results may not be accounted for. This review article aims to collect and evaluate what is currently known about the UPS-autophagy interaction with reference to specific proteins and microRNAs (miRNAs) that are known to be altered or activated in promoting growth of GBM, with the grand goal of finding potential targets for therapy-sensitization and induction of apoptotic cell death in GBM.

## 2. UPS Molecular Components and Functions in Cell Survival Show Therapeutic Opportunities in GBM

**Figure 1:**
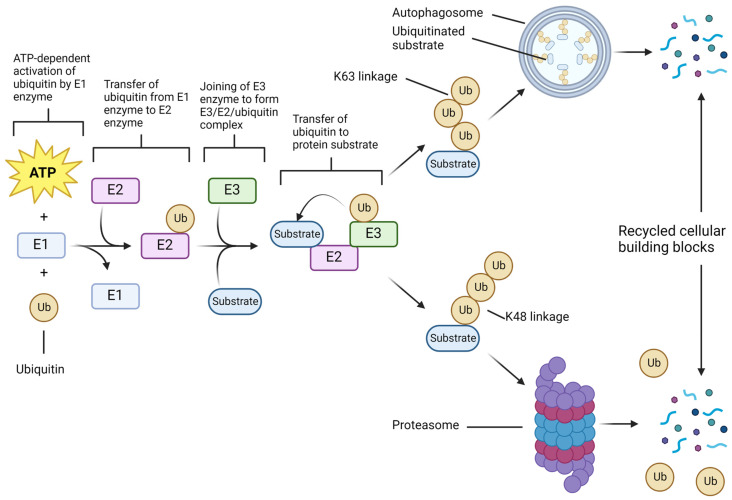
Ubiquitin plays an essential role in proteasomal and autophagic degradation of proteins in the GBM cells. Ubiquitin forms the basis of both autophagic and proteasomal signaling. Ubiquitination begins with the ATP-dependent activation of ubiquitin by an E1 enzyme. The activated ubiquitin is then passed to an E2 enzyme, which subsequently serves as a link between the target substrate and the E3 ubiquitin ligase. The E3 ligase then catalyzes the transfer of ubiquitin from the E2 enzyme to the substrate. Substrates may be mono-ubiquitinated, multi-mono-ubiquitinated, or have a ubiquitin chain attached; ubiquitin chains can be linked in a variety of ways including mixed and branched chains. Different configurations of ubiquitin linkages determine the pathway the substrate will continue down; this is known as the ubiquitin code. K48 linked chains tend to direct the substrate to proteasomal degradation, while K63 chains mark the substrate for autophagic degradation. Many other types of linkages are possible but are not shown here. Ub, ubiquitin.

Multiple studies have shown that GBM cells exhibit changes in the expression of genes associated with UPS [13,14]. Ubiquitin, a highly conserved protein with a length of 76 amino acids, is expressed in all cell types and ubiquitination, the attachment of ubiquitin to a substrate, is one of the most common post-translational modifications of proteins. Ubiquitin, originally noted for its function in the marking of proteins for degradation by the proteasome, was found to play a role in numerous other processes such as autophagy [10]. Ubiquitination occurs via a process involving three types of enzymes: E1, E2, and E3 (Figure 1). There are two E1 enzymes, which can facilitate the start of the ubiquitin signaling cascade: ubiquitin like modifier activating enzyme 1 (UBA1) and UBA6; other E1 enzymes exist but they initiate the conjugation of other ubiquitin-like proteins (UBLs) [15,16,17]. In an ATP-dependent manner, E1 enzymes bond to and catalyze the activation of free ubiquitin, which is then transferred to an E2 enzyme [15]. An E3 enzyme then transfers the ubiquitin to the final substrate. Really Interesting New Gene (RING) finger E3 enzymes act as a scaffold, bringing E2 and substrate into proximity and promoting a reaction where the E2 itself catalyzes the transfer of ubiquitin, whereas the Homologous to E6-AP Carboxyl Terminus (HECT), RING-Between-RING (RBR), and RING-Cys-Relay (RCR) families of E3 enzymes also bring together E2 and substrate, but catalyze the transfer themselves [18,19,20,21,22]. There are more than 40 E2 and more than 600 E3 enzymes. Specificity of substrate ubiquitination is largely determined by E3 enzymes as mediated by interactions with E2 and substrate [23]. Ubiquitination is a readily reversible process, and the enzymes that facilitate removal of ubiquitin from a substrate are known as deubiquitinases (DUBs). Ubiquitin signaling therefore depends on both the activities of ubiquitinating enzymes as well as DUBs. In addition, DUBs are responsible for the maintenance of the free ubiquitin pool by processing ubiquitin moieties from newly translated precursor proteins, as well as by removing ubiquitin from substrates prior to proteasomal degradation [24]. Additionally, the components of the ubiquitination cascade, as well as DUBs, may themselves be modified by ubiquitin or ubiquitin-like conjugation, adding additional complexity to their regulation [25,26,27]. Differential expression of E3 ligases and DUBs, such as ubiquitin specific protease 1 (USP1), USP7, Skp1-Cul1-F-box (SCF) ubiquitin ligase—the β-transducin repeat-containing protein (βTrCP) or SCFβTrCP, mouse double minute 2 homolog (MDM2)—also known as E3 ubiquitin-protein ligase, heat shock cognate protein 70 (HSC70)-interacting protein (CHIP), and many others has been linked to GSC stemness and invasiveness [6,28,29]. 

Substrates may be ubiquitinated in several ways that include conjugation of a single ubiquitin (mono-ubiquitination), conjugation of a single ubiquitin at multiple sites (multi-mono-ubiquitination), and linear or branched chains; in formation of chains, each ubiquitin has eight possible linkage sites for the attachment of a subsequent unit: seven lysine sites (K6, K11, K27, K29, K33, K48, and K63) and one methionine site (M1) [30,31,32,33]. Chains may be homotypic (same type of link throughout the chain) or heterotypic (multiple link types). Additionally, ubiquitin is itself subject to post-translational modifications including phosphorylation, deamidation, etc. [34]. The large number of possible configurations form a ubiquitin code, which can be read by ubiquitin-binding domains (UBDs) on downstream proteins to confer specificity in signaling; as very general examples, K48 chains are associated with proteasomal degradation, while K63 chains are linked to autophagy and DNA repair response [35]. 

While the degradation of large aggregates of protein and/or dysfunctional organelles is generally the domain of autophagy, the UPS is responsible for and focused on breaking down ubiquitinated soluble proteins. The 20S core particle (CP) of the eukaryotic proteasome is a barrel-shaped protein complex consisting of four stacked α and β hetero-heptameric rings, each ring consisting of subunits α1 through α7, or β1 through β7, and a central channel. Two stacked β-rings form the middle of the complex, with an α-ring on each end [36]. The proteolytic activity of the proteosome is dependent on the β-rings, specifically on subunits β1 (caspase-like), β2 (trypsin-like), and β5 (chymotrypsin-like) [37,38]. In the 20S proteasome, the α-rings are responsible for the recognition and binding of substrates, with the N-termini of α1–7 blocking the central channel until a suitable substrate is recognized, at which point the α subunits undergo a conformational change and the substrate is fed through to the proteolytic β-rings as a single polypeptide strand. The 20S proteasomes have been shown to, in an ATP-independent manner, preferentially degrade non-ubiquitinated proteins exhibiting an exposed unstructured region, though the exact mechanism of substrate recognition remains to be discovered [38]. After proteolysis in the central chamber located between the two β-rings, the products consisting of peptide chains with lengths ranging from 3 to 23 amino acids are released back into the cytosol. In GBM, overexpression of the epidermal growth factor receptor (EGFR) in the IR-resistant EGFR-III variant cells is linked to modified proteasomal composition and increased proteasome activity versus control cells after IR exposure, indicating a role for the UPS in conferring the IR resistance [39]. 

The CP of the proteosome may be modified in numerous ways, allowing its specificity to be modified rapidly in response to changing conditions in the cells. The most significant type of modification involves the addition of a regulatory particle (RP) activator to one or both ends of the CP. A single 19S RP ‘lid’ in association with the CP is the most common configuration, and together they form the 26S proteasome [7,40,41]. The 19S RP acts as a receptor for ubiquitinated proteins that have been marked for degradation, subsequently deubiquitinating and unfolding the substrate in an ATP-dependent manner and feeding it into the CP [42]. Structurally, the 19S RP contains the ‘base’ and ‘lid’ subcomplexes. The base contains six AAA-ATPases (Regulatory particle ATPase 1 to 6 or Rpt1–6), which facilitate substrate unfolding, and the Regulatory particle non-ATPase 1 (Rpn1), Rpn2, Rpn10, and Rpn13, which serve structural and ubiquitin recognition functions [41]. The lid contains Rpn3, 5–8, 9–12 and 15; it is primarily linked to deubiquitination of the substrate via the DUB Rpn11 in association with the non-integral RP-associated DUBs such as USP14 and ubiquitin C-terminal hydrolase 37 (UCH37) [43,44]. 

Other varieties of proteasome include the immunoproteasome, wherein the β1, β2, and β5 subunits of the CP are replaced by more efficient interferon-gamma (IFN-γ) inducible subunits termed β1i, β2i, and β5i, respectively. The immunoproteasome produces peptide fragments especially suited for recognition by the major histocompatibility complex (MHC) class I and subsequent CD8+ T cell response [45,46]. Differential immunoproteasome composition has been found in GBM cells and is correlated with decreased chymotrypsin-like activity [47]. The 20S proteasome activators (PAs), such as PA28αβ (also known as 11S) and PA28γ, are alternate caps which associate with the CP in a similar fashion to the 19S RP and act in an ATP-independent and ubiquitin-independent manner [48]. PA28αβ seems to activate all three types of proteolytic activity in the proteasome, while PA28γ is primarily found in the nucleus and it imitates the trypsin-like activity only, both exhibit unique peptide product profiles [48,49]. Another PA is PA200, which also associates with the CP in the same manner as PA28αβ/γ and 19S RP, and it functions independently of ATP and ubiquitin. PA200 is primarily localized in the nucleus and has been proposed to play a role in DNA repair mechanisms by targeting histones [48,50,51,52]. These alternative activators may associate with the CP singly, on both ends, and in conjunction with a 19S RP on the opposite end [46]. The large number of possible combinations therefore allows the proteasome to exhibit significant versatility and selectivity, though the physiological functions of many of these variations have yet to be elucidated.

The UPS acts as a major regulator of induction of apoptosis in cancers including GBM through both degradative and non-degradative ubiquitination of apoptosis related proteins, such as the anti-apoptotic Bcl-2 and the pro-apoptotic Bcl-2-interacting protein 1 (BNIP1) [53]. On the other hand, UPS can also promote cell survival by inhibiting pro-apoptotic proteins such as p53 and cytochrome c [53]. The proteasome inhibitor bortezomib used in combination with the apoptosis-inducing tumor necrosis factor (TNF)-related apoptosis-inducing ligand (TRAIL) has shown promising therapeutic effects in GBM due to stabilization of the pro-apoptotic protein tBid [54]. Another UPS-mediated strategy for treating GBM is the use of proteolysis targeting chimeras (PROTACs) to target specific proteins for proteasomal degradation. PROTACs utilize the UPS by linking a target protein with an E3 ubiquitin ligase to artificially induce the target’s ubiquitination and have been studied as inhibitors of cyclin-dependent kinase 4 (CDK4), CDK6, and histone deacetylase 6 (HDAC6)—all of which promote cell cycle progression in GBM and numerous other oncogenic proteins in other cancers [55].

## 3. Autophagy in the Degradation and Salvaging of Cellular Components to Support GBM Growth Indicating Potential Therapeutic Targets

**Figure 2:**
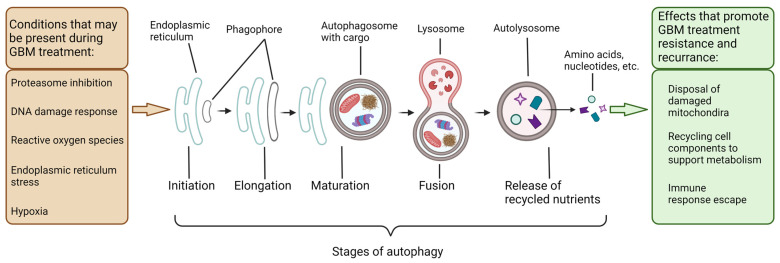
Conditions that lead to the inducement of autophagy in GBM and role of autophagy in treatment resistance in GBM. Conditions (e.g., hypoxia) that induce autophagy and are inherent to GBM, as well as those that may be conditional (e.g., proteasome inhibition), on specific treatments are documented here. The stages of autophagy include initiation of the phagophore at the omegasome, elongation of the double-layer phagophore membrane, sealing and maturation of the autophagosome, and fusion with the lysosome and subsequent cargo degradation with lysosomal acid hydrolases for recycling of the cellular building blocks being released back into the cytosol. Autophagy supports GBM cell survival by removing the damaged mitochondria before they can begin apoptotic signaling, mitigating the effects of hypoxia by recycling resources for the cell, and selective degradation of tumor suppressor proteins and immune signaling proteins.

There are three main types of autophagy: macroautophagy, chaperone-mediated autophagy, and microautophagy [56]. Macroautophagy (hereafter autophagy, unless mentioned otherwise) is the best characterized of the three types of autophagy and it is the one covered mainly in this review article. The process of autophagy begins at the omegasome, named for its resemblance to the Greek letter Ω, a cup-shaped structure in the endoplasmic reticulum (ER) membrane [57]. Subsequently, an extension of the ER membrane called the phagophore is formed and it begins to surround the cargo to be degraded [57]. In the case of non-selective autophagy, the phagophore encloses a volume of cytoplasm whose contents are essentially random; selective autophagy is dependent on numerous adapter proteins, which associate the targeted cargo with the inner surface of the phagophore [8]. Selective autophagy can target a wide variety of cargoes, such as protein aggregates, mitochondria (for mitophagy), peroxisomes (for pexophagy), foreign pathogens (for xenophagy), and more [56]. Once the phagophore has grown sufficiently its ends are sealed together to form a roughly spherical, double-membrane vesicle called an autophagosome [58]. The autophagosome and a lysosome are then directed together, and fusion occurs through the actions of several proteins, creating an autolysosome [59]. The contents of the autolysosome are then degraded by the acid hydrolases supplied by the lysosome and subsequently released back into the cytosol (Figure 2). 

Numerous studies have indicated that autophagy is dysregulated in GBM, and subsequently much research has been directed toward modulation of autophagy as a potential treatment strategy for GBM [5,60,61]. The understanding of the role of autophagy in GBM, as well as in other cancers in general, is rapidly evolving, and studies with seemingly contradictory results are not uncommon. The inter-tumoral and intra-tumoral heterogeneity prevalent in GBM can make a generalization very difficult, but some broad strokes on role of autophagy in GBM can be painted. 

In healthy brain cells, autophagy is thought to have a tumor suppressive effect, inhibiting the effects of DNA-damaging reactive oxygen species (ROS) by removing the damaged organelles and protein aggregates [62,63]. Beyond the early stages of tumorigenesis, autophagy is often found to be activated, with the level of activation negatively correlated with overall survival rate [64]. Autophagy can promote tumor growth by recycling cellular components to provide energy in hypoxic conditions, as well as by selective degradation of tumor suppressor proteins [62]. Autophagy has been proposed as a factor of epithelial-mesenchymal transition (EMT)/mesenchymal-epithelial transition (MET) and tumor invasiveness in GBM cells, although the exact mechanism is not currently known [65]. Because of its essential roles in tumor survival, growth, EMT/MET, and invasiveness, autophagy is a tempting target for inhibiting GBM in preclinical models.

Highly inhibited as well as highly activated autophagy can lead to cell death, and therefore all cells need to carefully regulate it [66,67,68]. However, this also means there are multiple paths to intentionally inducing cell death in tumors by tipping the scales in one direction or the other. Multiple studies have shown that the inhibition or activation of autophagy, either on its own or in combination with standard treatments like TMZ and/or IR, is more effective at inducing GBM cell death than TMZ and/or IR alone [60]. TMZ itself induces autophagy through the DNA damage response and ROS production, and this induced autophagy is thought to significantly contribute to the acquired TMZ resistance in GBM [69]. The effectiveness of autophagy modulation in one direction or the other likely depends on numerous factors including GBM type and the tumoral cell subpopulations in question [70]. However, the highly integrated role autophagy plays in tumor cell survival, metabolism, and genomic stability makes it an important consideration for the development of any therapies for controlling survival and growth of GBM.

## 4. Molecular Components in the Intersections of UPS and Autophagy Pose Challenges and Provide Therapeutic Opportunities in GBM

### 4.1. Ubiquitin-Specific Protease 14 (USP14) Acts in Promoting GBM Survival and Progression

**Figure 3:**
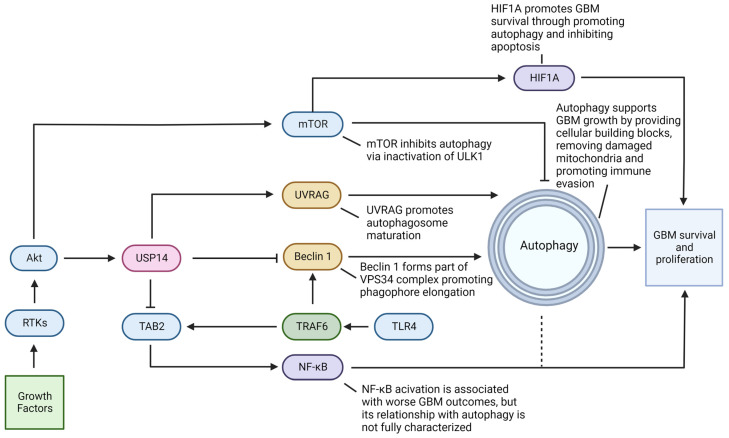
The USP14 regulation of pathways leading to autophagy and GBM survival. USP14 is a deubiquitinase, which directly regulates multiple autophagy-related proteins. USP14 is activated by Akt via phosphorylation, while Akt is a downstream effector of the receptor tyrosine kinases (RTKs), which are activated by growth factor receptors. USP14 antagonizes the E3 ligase tumor necrosis factor (TNF) receptor–associated factor 6 (TRAF6) by deubiquitinating and inactivating its substrates Beclin 1 and the transforming growth factor beta (TGFβ)-activated protein kinase 1 (TAK1)-binding protein 2 (TAB2). Beclin 1 is a critical pro-autophagy related protein while TAB2 is an activator of the nuclear factor-kappaB (NF-κB) signaling. USP14 also stabilizes the ultraviolet (UV) radiation resistance-associated gene (UVRAG) protein, which is known to support the maturation of autophagosomes. ULK1, Unc-51 like autophagy activating kinase 1; VPS34, vacuolar protein sorting 34.

USP14 is one of the three proteasome-associated DUBs, and it plays a central role in the regulation of both UPS and autophagy. USP14 is activated when it is associated with a proteasome through its UBL domain, and its regulation of proteasomal degradation is linked to its removal of the K48-linked ubiquitin chains from several substrates, as well as interactions with another proteasomal DUB, Rpn11 [71,72]. USP14 is also activated through phosphorylation by Akt; conversely, Akt is an inhibitor of autophagy through its promotion of the mechanistic target of rapamycin complex 1 (mTORC1) activity [73,74]. USP14 has been implicated in the tumorigenesis and progression of numerous cancers, often through its interactions with autophagy [75,76,77,78,79]. Similarly, USP14 has been shown to be highly overexpressed in GBM, causing cell survival and proliferation (Figure 3) [75,80,81]. However, in response to hypoxic conditions, GBM cells have been shown to express lower levels of USP14 mRNA [82]. Lung cancer cells treated with 6-Gingerol had reduced USP14 levels and increased levels of autophagy-associated proteins, leading to autophagy-dependent ferroptosis and tumor shrinkage in mice [83]. Similarly, future studies may indicate that pharmacological or genetic inhibition of transcriptional or translational expression of USP14 may constitute a therapeutic avenue leading to induction of cell death in GBM in vitro and in vivo.

USP14 has been shown to act as an activator of autophagy by deubiquitinating UVRAG, a component of the vacuolar sorting protein 34 (VPS34)/Beclin 1/UVRAG complex, which is critical for the proper fusion of autophagosomes and lysosomes [84]. The same study has also shown that proteasome activity is diminished upon induction of autophagy but activated upon inhibition of autophagy [84]. On the other hand, an earlier study indicated an inhibitory role for USP14 in the regulation of autophagy through the removal of the K63-linked ubiquitin chains on Beclin 1, inhibiting the formation of the VPS34/Beclin 1/ autophagy related gene 14 (ATG14) complex and suppressing autophagy at the stage of phosphatidylinositol 3-phosphate (PtdIns3P) production [85]. Additionally, an alternative mechanism for the inhibition of autophagy by USP14 has been suggested involving competitive binding of USP14 and Beclin 1 with tumor necrosis factor receptor-associated factor 6 (TRAF6), a downstream effector of the Toll-like receptor 4 (TLR4) signaling, which facilitates the K63-linked ubiquitination of Beclin-1. USP14 may also inhibit canonical nuclear factor-kappaB (NF-κB) signaling by deubiquitinating TAB2, another target of TRAF6 [86]. NF-κB and autophagy exhibit a two-way regulatory relationship, which is intricate and context-dependent [87]. 

The interactions between USP14 and autophagy also play a role in IR-induced DNA damage repair (DDR). USP14 inhibits non-homologous DNA end joining (NHEJ) and increases cell death in response to IR in phosphatase and tensin homolog (PTEN)-deficient prostate cancer cells through targeting several NHEJ-associated proteins, including Ku70, Ku80, and ring finger protein 168 (RNF168). Conversely, USP14 inhibition or PTEN overexpression restored NHEJ activity. Autophagy has been shown to negatively regulate USP14 through p62-mediated targeted degradation [88,89].

### 4.2. Etoposide Induced 2.4 Transcript (EI24) Acts as a Promoter of GBM Growth

EI24 is an important autophagy-associated protein and tumor suppressor that has been found to be under expressed in a variety of malignancies [90,91,92,93], although other studies have found no correlation or even positive correlation between EI24 expression and tumorigenesis in certain cancers, indicating that effects of EI24 depend on the cellular context of the cancers [94,95]. Additionally, EI24 regulates the UPS by targeting certain RING E3 ligases for autophagy mediated degradation; earlier studies have identified 161 RING E3 ligases as potential targets of EI24, the best characterized of which are TNFR-associated factor 2/5 (TRAF2/5), mouse double minute 2 homolog (MDM2), and tripartite motif containing 41 (TRIM41)—also called RING finger protein that interacts with C kinase 1 (RINCK1) [94,96]. 

High levels of TRAF2, an E3 ligase that plays a role in NF-κB signaling, have been found to be correlated with less favorable outcomes in GBM patients [97], potentially due to the promotion of tumor cell migration [98,99], radio-resistance [100], and stemness of GSCs and GBM initiating cells (GICs) [101]. TRAF2 inhibition has been shown to increase TNFα-induced apoptosis in GBM cells [102]. Additionally, TRAF2 may act as an activator of autophagy in response to ER stress through the TRAF2/inositol-requiring kinase-1 (IRE1)/C-jun NH_2_-terminal kinase (JNK) pathway, promoting Beclin 1/Bcl-2 dissociation [103]. 

The E3 ligase MDM2 regulates the important tumor-suppressor protein p53 via ubiquitination and proteasomal degradation [104]. In GBM, MDM2 gene amplification is present in 8.45 to 11% of cases and occurs exclusively for mutations/deletions of p53 gene [105,106]. Another significant player in this pathway is ubiquitin specific peptidase 7 (USP7, also known as the herpes virus associated ubiquitin-specific protease or HAUSP), a DUB that targets and stabilizes p53, MDM2, and MDM4 [107]. USP7 is upregulated in GBM, and its higher levels are correlated with lower survival rates [108], although the USP7 gene itself is not frequently altered in GBM [106]. In addition to its negative regulation of p53, MDM2 has been shown to promote GSC stemness and chemoresistance by tagging nucleolin, a putative inhibitor of GSC markers, for degradation [109]. Other p53-independent and possibly tumorigenic interactions of MDM2 have previously been described and include, among other effects, ubiquitination, and inhibition of forkhead box O3A (FOXO3A), E-cadherin, and Slug [110]. Many studies into MDM2 inhibitors as potential radiosensitizers and chemosensitizers for GBM therapy have already been carried out, although few candidates have moved past preclinical trials so far [111]. 

Some of the downstream targets of EI24 are well-known promoters of tumorigenicity in GBM, making it a potentially effective target for therapeutic modulation. However, its position as a major nodule of the UPS-autophagy crosstalk means modulation of its activity may have far-reaching effects on both systems. Unfortunately, there is limited information about the role of EI24 specifically in GBM, which in the light of conflicting reports of its action as a tumor suppressor in various tissues, leaves its suitability as a therapeutic target the subject of future research.

### 4.3. Hypoxia-Inducible Factor-1A/2A (HIF1A/2A) Facilitates GBM Growth and Recurrence

Hypoxia is well-known to regulate angiogenesis, metabolism, and therapeutic response in malignant tumors, including GBM. In fact, hypoxic conditions are very common and essential for GBM growth; despite the increased levels of angiogenic signals, such as vascular endothelial growth factor (VEGF), the vascular networks formed within the tumor are poorly connected and abnormal, resulting in tissue areas that are oxygen deprived and necrotic. Analysis of gene expression in hypoxic areas indicates that tumor cells adopt numerous adaptations to facilitate survival in this hypoxic environment [4]. Hypoxia itself inhibits the efficacy of many potential therapies for GBM, and the hypoxia-induced adaptations in these cell populations also help protect them from typical therapies such as IR and TMZ and may play a significant role in the development of treatment resistance and subsequent tumor resurgence—very typical of GBM [112,113].

HIF1A is considered the most significant regulator of hypoxic response in GBM, and it interacts and co-operates in a significant way with both UPS and autophagy for tumor growth [11,114]. In the case of normoxia, prolyl hydroxylase domain (PHD) enzymes hydroxylate HIF1A, leading to its ubiquitination by the E3 von Hippel–Lindau (VHL) protein and proteasomal degradation [115]. Hypoxic conditions inactivate PHDs, allowing HIF1A the opportunity to translocate to the nucleus and form a protein complex, which acts as a transcription factor for the genes critical to hypoxia response promoting tumor cell survival [115].

**Figure 4:**
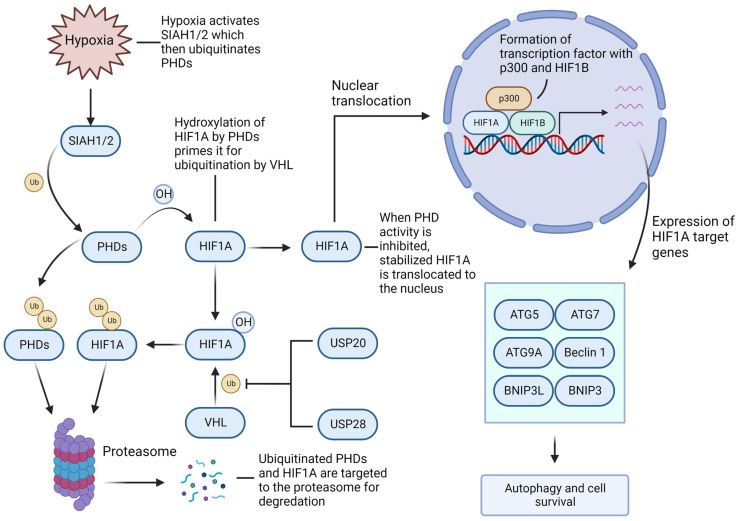
Effects of hypoxia on proteasomal and autophagic activity in GBM. Under normoxia, prolyl hydroxylase domain (PHD) enzymes hydroxylate HIF1A, priming it for ubiquitination by von Hippel–Lindau (VHL) protein and subsequent degradation by the proteasome. The deubiquitinases USP20 and USP28 stabilize HIF1A by reversing VHL-mediated ubiquitination. Under hypoxic conditions, the E3 ubiquitin ligase seven-in-absentia 1/2 (SIAH1/2) ubiquitinates PHDs and directs them to proteasomal degradation, stabilizing HIF1A to enter the nucleus and act as a transcription factor for various autophagy-related genes, which promote cell survival in GBM.

Hypoxia and autophagy work hand-in-hand in promoting cell survival in GBM (Figure 4). Hypoxia-induced HIF1A-mediated autophagy activation occurs through a pathway independent of, but complimentary to, the classical starvation-induced AMP-activated protein kinase (AMPK)/mTOR-mediated pathway, although in case of severe hypoxia both may function concurrently [116]. HIF1A promotes expression of core autophagy genes such as ATG5/7/9A, phosphatidylinositol-4,5-bisphosphate 3-kinase catalytic subunit alpha (PIKC3CA), Beclin 1, Bcl-2-Interacting Protein 3 (BNIP3), and BNIP3 Like (BNIP3L) [117]; the latter two do not have a direct role in autophagy but act to free Beclin 1 from two potent anti-apoptotic proteins, Bcl-2 and Bcl-xL, through competitive binding [118]. Additionally, the HIF1A isoform HIF2A was found to regulate the hypoxia response in GSCs, which were stabilized by USP33 [119].

The stability of HIF1A/2A is determined by a balance between the activity of UPS and several HIF1A/2A-targeting DUBs including USP20, USP28, and USP33 [119,120,121]. Additionally, PHDs are targeted for proteasomal degradation by the hypoxia-associated E3 seven-in-absentia 1/2 (SIAH1/2), indirectly promoting HIF1A stability [122,123]. Therefore, inhibiting the protective metabolic and autophagic responses under hypoxia, via the activation of proteasomal degradation of HIF1A/2A through the inhibition of HIF1A/2A-associated USPs and/or SIAH1/2, or by HIF1A/2A-targeting PROTACs, may be a promising avenue for sensitizing GBM cells to the existing and future therapies.

### 4.4. Histone Deacetylase 6 (HDAC6) Lends a Hand in Rapid GBM Progression

The HDAC family of enzymes facilitates epigenetic regulation of genes through the post-translational modification (i.e., removal of acetyl groups) of histones in nucleosomes, resulting in the compaction of chromatin and inhibition of transcription. HDACs have been shown to be overexpressed in many cancers, including GBM [124], and the overexpression of HDACs is associated with more rapid GBM progression [125]. HDACs have been found to be potentially oncogenic as they can reduce expression of the tumor suppressor genes; subsequently, HDAC inhibitors (HDACi) have been a subject of great interest in the treatment of cancers and other diseases. There already exist a few HDACi approved by the Food and Drug Administration (FDA) for some cancers (although not specifically for GBM) and several more HDACi are currently going through clinical trials [126]. There are 18 HDAC isoforms belonging to several classes, each with non-redundant functions and different cellular localizations, and all of these are expressed differentially in various tissues [127,128]; the following section will cover HDAC6, as it has a particular relevance to the UPS-autophagy axis, and development and treatment-resistance of GBM. 

HDAC6 has, in addition to its chromatin remodeling function, numerous non-histone substrates that it is able to act on due to its primarily cytoplasmic localization [129]. Recently, the transcription factor Specificity protein 1 (Sp1) was identified as a substrate of HDAC6; deacetylated Sp1 promotes the transcription of several oncogenes potentially related to TMZ resistance in GBM cells, and levels of both HDAC6 and deacetylated Sp1 were increased after long-term TMZ treatment [130]. The inhibition of HDAC6 results in activated mismatch repair (MMR) protein via MutS homolog 6 (MSH6) stabilization, as well as decreased levels of the epidermal growth factor receptor (EGFR), mutp53, O6-methylguanine-DNA methyltransferase (MGMT), and MDM2 in GBM cells, resulting in increased apoptosis when combined with TMZ treatment (Table 1) [131]. It has been shown that HDAC6 stabilizes EGFR and contributes to TMZ resistance in GBM cells, which is reversed when cells are concurrently treated with the HDAC6 inhibitor CAY10603 [132].

Another study using the HDAC6 inhibitors CAY10603 and ACY1215 found that the mitogen-activated protein kinase kinase 7 (MKK7)/JNK/c-Jun pathway was repressed by those inhibitors, suppressing the growth of GBM xenografts in mice [133]. Inhibition of HDAC6 using small interfering RNA (siRNA) technology has been shown to suppress several contributing aspects of GBM progression, including EMT, cell migration, proliferation, and autophagy [134]. The transactive response DNA binding protein 43 kDa (TDP-43) is well known to be a DNA and RNA binding protein that can be involved in RNA processing. TDP-43 promotes expression of HDAC6 and ATG7, subsequently inducing autophagy and GBM tumorgenicity; TDP-43 is itself regulated by ubiquitination and proteasomal degradation [135]. Furthermore, when UPS is impaired TDP-43 is known to form the cytotoxic sequestosome 1 (SQSTM1)/p62-positive aggresomes, which depend on autophagy for clearance [136,137]. The interaction between HDAC6 and the ATP-driven p97/chaperone valosin-containing protein (VCP) is another crossroads of the UPS-autophagy crosstalk and TMZ resistance in GBM. HDAC6 acts to clear ubiquitinated proteins by binding them with its UBP domain while simultaneously associating with the Dynein-Dynactin system, resulting in the delivery of the proteins to aggresomes and subsequent degradation by autophagy [138]. As stated above, p97/VCP is a chaperone protein complex that promotes proteasomal degradation of the ubiquitinated proteins; however, it can also compete with the ubiquitinated proteins in binding to HDAC6, and therefore inhibit the HDAC6-mediated autophagic clearing of the misfolded proteins [139]. In GBM cells, the ratio of HDAC6 to p97/VCP is larger than normal, contributing to increase in aggrephagy, the 78 kDa glucose-regulated protein 78 (GRP78)-dependent ER stress tolerance, and TMZ resistance, which can be reversed in the presence of the HDAC6 inhibitor Tubastatin A [140]. 

### 4.5. Phosphatase and Tensin Homolog (PTEN)-Induced Kinase 1 (PINK1) and Parkin Act as Tumor Suppressors in GBM

**Figure 5:**
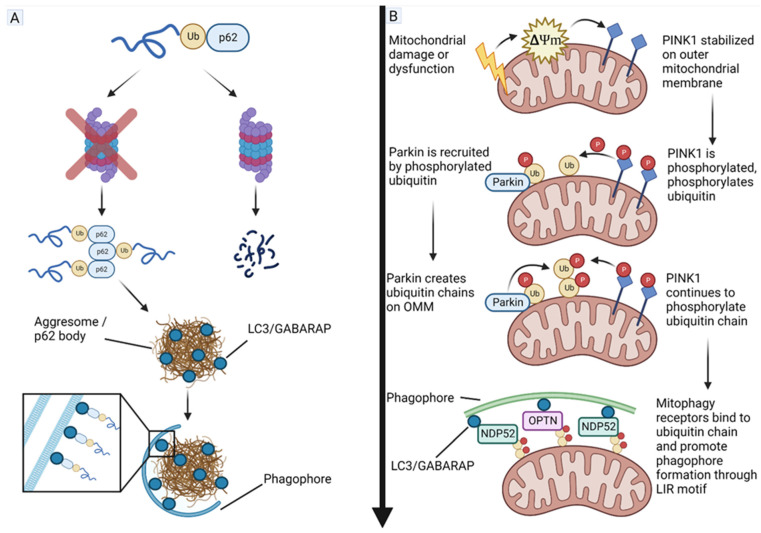
Interactions between the UPS and selective autophagy in GBM. (**A**) Ubiquitinated proteins destined for degradation are bound by SQSTM1/p62. If UPS is functioning at a sufficient level, SQSTM1/p62 will act as a chaperone guiding the substrate into the proteasome. If UPS function is impaired, substrate-bound SQSTM1/p62 will oligomerize forming an aggresome or SQSTM1/p62 body in the cytosol. The SQSTM1/p62 body is joined by microtubule-associated proteins 1A/1B light chain 3B (hereafter referred to as LC3)/gamma-aminobutyric acid receptor-associated protein (GABARAP) via its LC3-interacting region (LIR) motif, which subsequently attracts the autophagy machinery to facilitate degradation. Protein aggresomes can become cytotoxic if allowed to accumulate. (**B**) Selective autophagy of mitochondria (mitophagy) is normally active at a low level in most cells but becomes activated when mitochondria become damaged (such as mtDNA damage from radiation) or otherwise dysfunctional. Mitochondria respond to damage through disruption of mitochondrial membrane potential (ΔΨm), allowing PTEN-induced kinase 1 (PINK1) to accumulate on the outer mitochondrial membrane (OMM). PINK1 proceeds to phosphorylate ubiquitin (Ub), attracting Parkin (a 465-amino acid residue E3 ubiquitin ligase), which is then also phosphorylated and activated. Parkin then begins building ubiquitin chains on OMM proteins, which PINK1 continues to phosphorylate. The phosphorylated ubiquitin chains then recruit mitophagy receptors including nuclear dot protein 52 (NDP52) and optineurin (OPTN). These mitophagy receptors then bind to LC3/GABARAP via their LC3 interacting region (LIR) motif. LC3/GABARAP then recruits autophagy initiation machinery such as the Unc-51 Like autophagy activating Kinase 1 (ULK1) to begin forming a phagophore around the cargo leading to degradation.

Normal metabolism is significantly altered in GBM and many other solid tumors; these changes primarily occur as a response to hypoxia and/or mutations altering gene expression/protein function. A common alteration, called the Warburg effect, is characterized by the preferential redirection of pyruvate into the fermentative pathway instead of its utilization for oxidative phosphorylation (OXPHOS), even in an abundance of oxygen (aerobic glycolysis) [141]. However, OXPHOS is not completely shut down and the Warburg effect, and the ratio of ATP derived from aerobic glycolysis to that from OXPHOS, varies between cancer types, especially with reference to the heterogenous nature of GBM tumors, and between subpopulations of cells within the same tumor [141]. GSCs, which are the main suspects when it comes to GBM tumor recurrence, have been shown to utilize aerobic glycolysis while also maintaining OXPHOS at high levels compared to differentiated GBM cells; this may be a characteristic which leads to their increased resistance to treatment [142,143]. Therefore, management of the Warburg effect and mitochondrial dynamics may be crucial for controlling GBM growth and recurrence. Studies have shown interactions between the UPS and selective autophagy (mitophagy) that play critical roles in promoting survival and growth of GBM cells (Figure 5).

The PINK1 protein is a linchpin in mitochondrial quality control through selective autophagy of mitochondria, called mitophagy. PINK1 is under expressed in GBM while GBM tumor growth and the Warburg effect have been shown to be partially dependent on PINK1 expression [144]. PINK1 is translated in the cytoplasm and transported across the outer mitochondrial membrane (OMM) and the inner mitochondrial membrane (IMM), where it is cleaved by mitochondrial processing peptidase (MPP) and presenilin-associated rhomboid-like protease (PARL), and subsequently returned to the cytoplasm where, under normal conditions, it is either degraded by the proteasome or stabilized by a chaperone protein, exerting an inhibitory effect on mitophagy through interactions with Parkin (an ubiquitin E3 ligase that mono-ubiquitinates and poly-ubiquitinates proteins) and protein kinase A (PKA) [145]. There is also evidence that cytosolic PINK1 acts as a sensor of the UPS stress as its accumulation upon proteasome inhibition results in the PINK1-dependent phosphorylation of the translation elongation factor, known as the eukaryotic translation elongation factor 1 alpha 1 (eEF1A1), leading to decreased translational activity [146]. Upon mitochondrial damage or dysfunction, the mitochondrial membrane potential is decreased, causing PINK1 to remain on the OMM where it is dimerized and becomes phosphorylated [147]. The OMM-bound PINK1 then proceeds to phosphorylate various proteins such as Mitofusin 2 (Mfn2), Miro, Bcl-xL, and Drp1; additionally, it phosphorylates ubiquitin, which is required for the recruitment of Parkin [148]. PINK1 is a potential tumor suppressor in GBM, and its overexpression may form a therapeutic strategy for inhibiting growth of GBM cells.

As mentioned above, Parkin is an E3 ubiquitin ligase, which is recruited to the OMM by the phosphorylated ubiquitin and subsequently activated by PINK1 via phosphorylation. Like PINK1, Parkin is down regulated in GBM, and its expression is positively associated with more favorable prognosis [149,150]. Once activated, Parkin ubiquitinates OMM-bound proteins, recruiting mitophagy receptors, such as nuclear dot protein 52 (NDP52) and optineurin (OPTN), via their ubiquitin-associated (UBA) and ubiquitin-binding zinc finger (UBZ) domains, respectively; these proteins also contain the microtubule-associated protein 1A/1B light chain 3B (referred to as LC3) interacting region (LIR) motifs, which promote the formation of the phagophore around the mitochondria, ultimately leading to autophagic degradation [151,152]. Parkin has been shown to downregulate HIF1A via proteasomal degradation in breast cancer leading to inhibition of its metastasis [153], and similarly Parkin has been reported to downregulate HIF1A while upregulating HIF3A in GBM [154]. Parkin also has a role in the regulation of the cell cycle through down regulating cyclin E [155] while upregulating cyclin dependent kinase 6 (CDK6) [156], both leading to inhibition of cell cycle progression. Parkin is in turn regulated by several DUBs; USP8 promotes Parkin activity and mitophagy by removing the K6-linked ubiquitin chains, which prevent Parkin’s interaction with the phosphorylated ubiquitin and PINK1 [157], while USP15 and USP30 antagonize Parkin by removing ubiquitin chains from mitochondria, preventing the binding of mitophagy receptors [158,159]. A recent study suggested that endogenous Parkin acted as a potential tumor suppressor and its overexpression could block cell cycle via trans-repression of cyclin A and cyclin B genes for controlling growth of GBM cells [160].

### 4.6. microRNAs (miRNAs) and Their Roles in Promoting or Inhibiting GBM Growth

The miRNAs are short (19–25 nt) RNA transcripts, which target the 3′ end of mRNAs with complimentary sequences so as to inhibit their translation into proteins by either destabilizing the mRNA by deadenylating its poly(A) tail or by marking the mRNA for cleavage by the RNA-induced silencing complex or RISC [161,162]. Alteration of miRNA expression has been associated with numerous pathological conditions, including cancer, aging, cardiovascular disease, neurodegenerative diseases, and more [162,163,164,165,166]. miRNAs are in turn regulated by circular RNAs (circRNAs), long non-coding RNAs (lncRNAs), and pseudogenes that act as ‘sponges’ directing miRNAs away from their targets [167]. Analysis of miRNA targets indicates many of the human proteins are subject to miRNA-based regulation [168], and since their discovery research into therapeutic utilization of miRNAs has been significant [169,170].

GBM exhibits a unique miRNA expression profile [171,172], presenting opportunities for highly targeted therapies effecting pathways and processes tailored to the unique conditions present in GBM tumors through the introduction of exogenous miRNA or miRNA sponges with the aim of inhibiting tumor growth and inducing sensitivity to conventional treatments. However, significant difficulties still exist in the implementation of miRNA-based therapies especially concerning their delivery, and this is exacerbated in the case of GBM due to the presence of BBB, although efforts to overcome this issue are ongoing [173]. In the light of the significant advancements being made in miRNA research, this section will cover some miRNAs, which affect the UPS-autophagy axis with the potential of sensitizing GBM to IR, TMZ, and other treatments. 

The oncogenic miR-21 has been found to be overexpressed in GBM tumor cells, as well as in the cerebrospinal fluid of GBM patients [174,175], and it is negatively associated with patient survival [176]. miR-21 has been tied to the regulation of numerous pathways governing GBM tumorigenesis and progression, including UPS and autophagy. For example, IR induces miR-21 expression while inhibition of miR-21 in IR-treated GBM cells leads to autophagy-associated cell death [177]. Tamoxifen (an estrogen receptor antagonist) treatment leads to increased cell death when combined with miR-21 inhibition in GBM cells, potentially through inhibition of Bcl-2, an inhibitor of apoptosis [178]. In a similar vein, miR-21 inhibition in breast cancer cells treated with tamoxifen or fulvestrant (an estrogen receptor antagonist) lead to autophagic cell death through down regulation of the PTEN-Akt-mTOR pathway [179]. On the other hand, miR-21 levels are increased in multiple myeloma cells when the proteasome 20S subunit beta type-4 (PSMB4) is overexpressed; there is evidence that this is due to increase in proteasomal degradation of the inhibitor of NF-κB, as NF-κB promotes the transcription of miR-21 [180]. However, miR-21 has been shown to be an inhibitor of several proteasomal subunits, indicating a possible negative feedback mechanism [181]. Additionally, in pancreatic cancer cells, miR-21 is involved in the hypoxia response by targeting von Hippel–Lindau (VHL) factor, preventing the ubiquitination of HIF1A and its degradation by UPS [182].

Another miRNA frequently dysregulated in GBM is miR-93 [183]. miR-93 modulates autophagy in GBM by altering expression of several autophagy-related proteins such as ATG4B, ATG5, Beclin 1, and SQSTM1/p62, leading to the increased IR and TMZ sensitivity of GSCs [184]. Importantly, the effect of miR-93 modulation was found to depend on the differential endogenous expression levels of miR-93 in different GBM subtypes (proneural vs. mesenchymal) [184]. It has been reported that miR-93 also targets ATG12 to sensitize colorectal cancer to IR [185]. Additionally, miR-93 has been shown to regulate autophagy under hypoxic conditions by targeting ULK1, while miR-93 is down regulated under hypoxic conditions [186]. miR-93, in conjunction with miR-106b, also negatively regulates mitophagy induced by oxidative stress by targeting NDP52, mitochondrial calcium uniporter (MCU), Mfn2, and OPTN [187]. On the other hand, miR-93 influences the UPS by decreasing the neural precursor cell expressed developmentally downregulated gene 4-like (NEDD4L), an E3 ubiquitin ligase, which inhibits transforming growth factor beta (TGFβ) signaling in lung cancer cells by marking the mothers against decapentaplegic homolog 2/3 (SMAD2/3) proteins for proteasomal degradation. Lower levels of NEDD4L have been linked to higher grade astrocytomas and faster tumor progression [188]. Another study reports that in breast cancer cells, expression of the miR-106b-93-25 cluster results in NEDD4L down regulation and subsequent stabilization of Notch1, a direct target of NEDD4L ubiquitination [189]; Notch1 is generally overexpressed in GBM and is linked to lower overall survival [190].

## 5. Conclusions

GBM remains a particularly intractable malignancy in humans. A lack of effective new treatments indicates in recent years the need for novel strategies for tackling the challenges that GBM presents. Therapies involving modulation of either UPS or autophagy have been investigated, and some are in clinical trials; however, no new standard treatments have yet emerged from these therapeutic candidates. The frequent failure of the proposed new GBM therapies in clinical trials is due to multiple factors. Besides the factors (e.g., inter-tumoral and intra-tumoral heterogeneity, difficulty of full resection, and the presence of BBB) that make GBM inherently difficult to treat, flaws in laboratory reproduction of the tumor structure and microenvironment also impede translation to clinical results. The high rate of failures in Phase III clinical trials has also been attributed to insufficient control arms in the Phase II clinical trials they are based on [2]. The detailed analysis of the outcomes of the recent clinical trials in GBM management lies outside of the scope of this article, but there are recent in-depth reviews on this topic [191,192]. Because of their closely interrelated nature and integration into cell survival pathways, we propose that targeting UPS and autophagy simultaneously by modulating factors that act as centers of crosstalk between these two cellular recycling systems may open the window of promising opportunities for reducing treatment resistance and inducing apoptotic cell death in GBM, leading to the improved outcomes. A two-pronged approach such as this may be more effective at mitigating the compensatory functions that can arise when just one of these systems is targeted. Development of future therapies involving the UPS-autophagy interaction will benefit from research into further identification and characterization of ubiquitin ligases and DUBs, which determine what proteolytic pathway a substrate will enter, as well as increased understanding of more direct interactions, such as proteaphagy and proteasome-mediated degradation of ATG proteins. In addition, there is evidence of E3 ligases and DUBs interacting with UPS and autophagy pathways in ways that are independent of their direct interaction with ubiquitin, adding another layer of complexity yet to be fully elucidated for exploring new treatments for controlling survival and proliferation of GBM.

## Figures and Tables

**Table 1:** HDAC6 inhibitors and their targets for controlling growth of GBM.

HDAC6 Inhibitor	Downstream Target(s)	Effects on GBM	References
MPT0B291	Sp1	Decreased transcription of BMI1, hTERT, and cell cycle genes	[130]
A452	EGFR MGMT MSH2 MSH6 mutp53	Increased apoptosis when combined with TMZ	[131]
ACY-1215	EGFR MGMT MSH2 MSH6 mutp53 MKK7/JNK/c-Jun	Increased apoptosis when combined with TMZ causing decrease in tumor growth	[131,132,133]
CAY 10603	EGFR MGMT MSH2 MSH6 mutp53 MKK7/JNK/c-Jun	Increased apoptosis when combined with TMZ causing decrease in tumor growth	[131,132,133]
Tubastatin A	MSH2 MSH6 mutp53 p97/VCP	Increased apoptosis when combined with TMZ	[131,132]
siRNA (SASI_Hs01_ 00048982 and SASI_Hs02_0034079)	α-Tubulin Slug Snail Sonic Hedgehog	Decreased proliferation and migration, and reverted EMT phenotype	[134]

BMI-1, B lymphoma Mo-MLV insertion region 1 homolog; hTERT, human telomerase reverse transcriptase
